# AMP-Activated Protein Kinases in Health and Disease

**DOI:** 10.3390/ijms26168075

**Published:** 2025-08-21

**Authors:** Hyo In Kim, Yohan Han, Jinbong Park

**Affiliations:** 1 Department of Surgery, Beth Israel Deaconess Medical Center, Harvard Medical School, Boston, MA 02215, USA; 2 Institute of Wonkwang Medical Science and Department of Microbiology, Wonkwang University School of Medicine, Iksan 54538, Republic of Korea; 3 Department of Pharmacology, College of Korean Medicine, Kyung Hee University, Seoul 02447, Republic of Korea

AMP-activated protein kinase (AMPK) is a highly conserved serine/threonine kinase that serves as a central metabolic sensor and regulator of energy balance at both the cellular and organismal levels [1]. In response to energy stress, reflected by an increased adenosine monophosphate (AMP)/adenosine triphosphate (ATP) or adenosine diphosphate (ADP)/ATP ratio, AMPK is activated through upstream kinases such as liver kinase B1 (LKB1) or calcium/calmodulin-dependent protein kinase kinase 2 (CAMKK2) and subsequently initiates a coordinated cellular program to restore energetic homeostasis [2,3]. This process is accomplished by switching off ATP-consuming anabolic processes and concurrently turning on ATP-generating catabolic pathways, including fatty acid oxidation, glucose uptake, and mitochondrial biogenesis [4]. AMPK achieves this by directly phosphorylating several key substrates, including acetyl-CoA carboxylase (ACC), TBC1D1, and peroxisome proliferator-activated receptor gamma coactivator 1-alpha (PGC-1α), which play critical roles in regulating its metabolic and mitochondrial effects [5]. Beyond this canonical role in energy metabolism, AMPK is also involved in diverse physiological processes such as autophagy [6], redox regulation [7], inflammation [8], and circadian biology [9]. In metabolically active tissues, including skeletal muscle [10], liver [11], and adipose tissue [12], AMPK activation regulates substrate utilization and insulin sensitivity. In immune cells, AMPK modulates inflammatory phenotypes and cellular plasticity [13,14], while in neurons, it influences synaptic remodeling and neuroprotection [15]. Given its broad regulatory spectrum, AMPK has emerged as a promising therapeutic target for a variety of diseases characterized by metabolic derangement and chronic stress. These include, but are not limited to, type 2 diabetes, obesity, non-alcoholic fatty liver disease, atherosclerosis, heart failure, neurodegenerative disorders, and cancer. Notably, pharmacological AMPK activators have demonstrated their effects in ameliorating the pathophysiological features of various diseases [16]. Several AMPK agonists, including metformin [17] and AICAR [18], have been widely studied. Additionally, more selective compounds such as PF-06409577 [19] and A-769662 [20], which selectively activate AMPK complexes containing the β1 subunit, have undergone preclinical or early-phase clinical evaluation for metabolic disorders [21,22]. Although their effects are generally considered less prominent than those of activators, AMPK inhibitors such as Compound C have also been explored in various disease contexts, including cancer, where the suppression of AMPK signaling alters cellular metabolism and pathological progression [23,24].

Despite these advances, key questions remain unresolved. The mechanistic pathways underlying its tissue-specific effects and the interplay with parallel signaling pathways such as mammalian target of rapamycin (mTOR) [25], sirtuins [26,27], and nuclear factor kappa-light-chain-enhancer of activated B cells (NF-κB) [28] continue to be active areas of investigation. Therefore, ongoing research is essential to fully understand the actions of AMPK, and to translate this knowledge into clinically viable interventions. In this Special Issue ‘AMP-Activated Protein Kinases in Health and Disease’, three original research articles and two comprehensive reviews illustrate both the depth and breadth of AMPK-centered research. Collectively, these studies offer novel insights into AMPK signaling in metabolic regulation, cardiovascular function, cancer biology, and systemic wasting conditions.

In their study on Muscovy ducks, Zhao et al. [29] identify gender-based differences in muscle development that are partially mediated by differential AMPK activity. By integrating phenotypic traits, hormonal profiling, and transcriptomic analysis, the authors show that estradiol-mediated suppression of phosphorylated AMPK in female ducks correlates with reduced expression of lipid metabolic genes such as CD36 and carnitine palmitoyltransferase 1A (CPT1A). This study highlights AMPK as a mechanistic link between sex hormones and muscle growth potential in avian species. Hernández-Serda et al. [30] investigate the cardiometabolic role of AMPK under hypoxic stress in a high-salt diet-induced hypertensive rat model. The authors report that novel antihypertensive compounds (LQM series) restore hypoxia-induced glucose uptake in cardiomyocytes through glucose transporter 1 (GLUT1) and 4 trafficking. Notably, in silico analyses suggest that the cardioprotective effects of these agents may be mediated by their interaction with the γ-subunit of AMPK, pointing to an unanticipated mechanism of action that couples blood pressure control with energy homeostasis. In the yeast model system, Simpson-Lavy and Kupiec [31] provide a unique evolutionary perspective on AMPK regulation. They demonstrate that the poly-histidine tract of the yeast AMPK ortholog Snf1 coordinates carbon metabolism and iron homeostasis via pH-sensitive interactions with the iron-regulatory factor Aft1. This nuclear-specific inhibition of Snf1 under iron-limiting conditions suggests a spatially constrained regulatory mechanism that ensures mitochondrial competency before nuclear metabolic reprogramming, which demonstrates the key role of AMPK in resource allocation and organelle communication.

Two review articles broaden the scope of this Special Issue. Kovale et al. [32] explore the crosstalk between AMPK and autophagy in cancer stem cells (CSCs). As CSCs rely on metabolic reprogramming and dormancy for survival and therapeutic resistance, autophagy emerges as a double-edged sword. AMPK, as a key autophagy regulator, enables CSC adaptation in nutrient-poor environments, emphasizing the need for strategies that can selectively disrupt AMPK-autophagy signaling in CSCs while sparing normal stem cells. Han et al. [33] review the role of natural products in cancer-induced cachexia, a complex metabolic syndrome involving severe muscle and adipose tissue wasting. This review article systematically summarizes previous reports on natural compounds shown to ameliorate cachexia and outlines the involvement of AMPK in their therapeutic effects. Importantly, as AMPK remains a pivotal node linking inflammation, energy metabolism, and tissue preservation, further investigation involving greater mechanistic clarity and translational validation using robust animal models is required.

Despite the diversity of models and methodologies, a consistently observed theme throughout the research is the central role of AMPK in maintaining homeostasis under metabolic, inflammatory, and environmental stress conditions. Taken together, the contributions in this Special Issue emphasize AMPK’s multidimensional regulatory capacity across cell types, organ systems, and disease states. From avian physiology to cardiovascular therapeutics, from unicellular yeast to complex oncological settings, AMPK continues to reveal its integrative function in health and disease. These studies not only reinforce AMPK as a promising drug target but also illuminate the necessity of disease- and tissue-specific contextualization in AMPK-directed therapies. As the Guest Editor Team, we express our sincere appreciation to the contributing authors, reviewers, and editorial team. It is my hope that the work presented here will stimulate further research into AMPK’s translational potential and accelerate the development of targeted interventions that harness its unique biological versatility. Future investigations should also consider sex-specific responses, long-term safety of chronic AMPK activation, and tissue-selective delivery mechanisms to maximize therapeutic efficacy while minimizing adverse effects.
